# Phase change for the accuracy of the median value in estimating divergence time

**DOI:** 10.1186/1471-2105-14-S15-S7

**Published:** 2013-10-15

**Authors:** Arash Jamshidpey, David Sankoff

**Affiliations:** 1 Department of Mathematics and Statistics, University of Ottawa, 585 King Edward Avenue, Ottawa, Canada, K1N 6N5

## Abstract

We prove that for general models of random gene-order evolution of *k *≥ 3 genomes, as the number of genes *n *goes to ∞, the median value approximates *k *times the divergence time if the number of rearrangements is less than *cn*/4 for any *c <*1. For some c* ≥ 1, if the number of rearrangements is greater than c*n/4, this approximation does not hold.

## Introduction

The iterative improvement of approximate solutions to the Steiner tree problem by optimizing one internal vertex at a time has a substantial history in the "small phylogeny" problem for parsimony-based phylogenetics, both at the sequence level [1] and the gene order level [2]. It has been generalized to iterative local subtree optimization methods such as "tree-window-hill" [3] and "disc covering" [4,5]. Here we focus on the "median problem" for gene order where we estimate the location of a single point (the median) in a metric space given the location of the three or more points connected to the median by an edge of the tree. Given *k *≥ 3 signed gene orders *G*_1_, ..., *G_k _*on a single chromosome or several chromosomes, and a metric *d *such as breakpoints [6], inversions [7], inversions and translocations [8], or double-cut-and-join [9], find the gene order *M *such that $\sum_{i=1}^{k}d\left(G_{i},M\right)$ is minimized.

Although it plays a central role in gene order phylogeny, the median suffers from several liabilities. One is that it is hard to calculate in most metric spaces. Not only is it NP-hard [10], but exhaustive methods are costly for most instances, namely unless $G_{1}\ldots \;,G_{k}$ are all relatively similar to each other, which we will refer to generically as the *similar genomes condition*. Another problem is that heuristics tend to produce inaccurate results unless a suitable similar genomes condition holds [11]. Still another, is the tendency in some metric spaces to degenerate solutions [12] unless the same conditions prevails.

In this paper we add to this litany of difficulties by showing that as *k *genomes evolve over time, as modeled by any one of several biologically-motivated random walks, there is a phase change after *n*/4 steps, where *n *is the number of genes. With *u < n*/4 steps, the sum of the normalized distances $\sum_{k}d/n$ from each of the genomes to the starting point - the ancestor - converges to *ku/n *in probability, and this is the median value. When u > c*n/4 steps, for a constant c* ≥ 1, the sum of the normalized distances to the median converges in probability to a value less than *ku/n*, and that the ancestor is no longer the median.

Our proof is inspired by a result of Berestycki and Durrett [13] in showing that the reversal distance between two signed permutations converges in probability to the actual number of steps, after rescaling, if and only if *u < n*/2. The technique is to construct a graph with genes as vertices and edges added between vertices according to how they are affected by transpositions. Properties of the number of components of random Erdös-Renyi graphs can then be invoked to prove the result.

## Definitions

We represent a unichromosomal genome by a signed permutation, where the sign indicates whether the gene is "read" from left to right (tail-to-head) or from right to left (head to tail) on the chromosome. Let $S_{n}^{\pm }$ be the signed symmetric group of order *n*, i.e. the space of all signed permutations of length *n*. A *reversal *operation applied to a signed permutation reverses the order, and changes the signs, of one or more adjacent terms in the permutation. A DCJ operation, which can apply not only to signed permutations but to more general genomes containing linear and circular chromosomes, *cuts *the genome in two places and rejoins pairs of the four "loose ends" in one of two possible new ways (one of which may be equivalent to a reversal). We define the reversal and DCJ distances, *d_r _*and *dcj*, to be the minimum number of reversal and DCJ operations, respectively, needed to transform one genome to another.

The *breakpoint graph BP*(Π, Π') of two genomes represented by Π and Π' contains vertices for the head and tail of each gene, *black edges *edges defined by the adjoining heads or tails of two adjacent genes in the genome Π and *grey edges *defined by two adjacent genes in the genome Π'. Let *id *= *I*, the identity permutation, and *BP*(Π) = *BP*(Π, *id*). It is well-known that

(1) dcj(Π)=n+1-|cBP(Π)|.

We need to define an orientation for grey (and black) edges of *BP*(Π). We traverse a cycle *c *∈ *cBP*(Π) in a counter-clockwise manner if we start at the left-most vertex of *BP*(Π) (in the usual representation), travel along its unique adjacent black edge and end at the same vertex through its unique adjacent grey edge. Then we say a black edge in *c *is positively oriented if we move along it from left to right in a counter-clockwise traversal. Otherwise we say it is negatively oriented. Similarly, for the grey edge (*i_t_*, (*i *+ 1)*_h_*) we say it is positively oriented if during a counter-clockwise traversal we move along it from *i_t _*to (*i *+ 1)*_h_*. Otherwise it is negatively oriented. We define the orientation function *ξ *on the edges of *BP*(Π) to be:

(2) $$\xi \left(e\right)=\left\{\begin{array}{cc} +1 & \text{if }e\;\text{is positively oriented}\\ -1 & \text{if }e\;\text{is negatively oriented}\text{.} \end{array}\right)$$

We say the black (grey) edges *e*, *e*' are parallel, denoted by *e *|| *e*' if *ξ*(*e*) = *ξ*(*e*'). Otherwise we say they are crossing. This is just a reformulation of Hannenhalli and Pevzner's original concept of oriented cycles. An oriented cycle in this definition is a cycle including at least one positively and one negatively oriented black edge. The mechanism by which a reversal affects a genome can easily be seen using the *BP *graph. Let *ρ *be a reversal acting on two black edges *e*, *e*' in *BP*(Π). If they are in two different cycles we have a merger of the two to construct a new cycle. But if *e*, *e*' are in a same cycle, that cycle either splits, if *e *∦ *e*', or does not split if *e *|| *e*'.

## Limit Behavior of the Median Value

Suppose *d^n ^*be a metric on the space of all signed permutations length *n*. For a set *A *of these permutations, define

(3) $$g_{A}^{d,n}:S_{n}^{\pm }\to {\mathbb{N}}_{0}=\mathbb{N}\cup \left\{0\right\},$$

(4) $$g_{A}^{d,n}\left(x\right):=\underset{y\in A}{\sum }d^{n}\left(x,y\right).$$

Then let

(5) $$m^{d,n}\left(A\right):=min\left\{g_{A}^{d,n}\left(x\right):x\in S_{n}^{\pm }\right\}.$$

*m^d,n^*(*A*) is called the median value of A under the metric *d^n^*. A signed permutation which makes $g_{A}^{d,n}$ minimum is called a median solution of *A*. Denote by *d_r _*and *dcj *the reversal and DCJ distances on $S_{n}^{\pm }$.

Let *X*_0 _= *id*, the identity permutation, and let $X_{t}^{n}$ be a stochastic process on $S_{n}^{\pm }$, where at random Poisson times *τ_κ_*, with rate 1, we choose two elements of $X_{\tau_{\kappa }}^{n},$ namely *i*, *j *and let *ρ*(*i*, *j*) operate on $X_{\tau_{\kappa }}^{n},$ that is

(6) $$X_{\tau_{\kappa }^{+}}^{n}=X_{\tau_{\kappa }}^{n}o\;\rho \left(i,\;j\right),$$

where *ρ*(*i, j*) is the reversal acting on *i *and *j*. We call $X_{t}^{n}$ a reversal random walk (r.w.) on. $S_{n}^{\pm }.$ Suppose $X_{t}^{1,n},\;\ldots ,\;X_{t}^{k,n}$ be *k *independent reversal r.w. all starting at the identity element, *id*. Define

(7) $$A_{t}^{\left(n\right)}:=\left\{X_{t}^{1,n},\;\ldots ,\;X_{t}^{k,n}\right\}$$

and

(8) $$\varepsilon_{t}^{d,n}:=g_{A_{t}}^{d,n}\left(id\right)-m^{d,n}\left(A_{t}\right).$$

We investigate the time up to which the median value of $X_{t}^{1,n},\;\ldots ,\;X_{t}^{k,n}$, namely *m^d,n ^*(*A_t_*), remains a good estimator for the total divergence time, *kt*, as well as to the total distance of points in *A_t _*to *id*, namely $g_{A_{t}}^{d,n}\left(id\right)$. To answer this question we use the fact that the speed of escape of the r.w. up to some particular time, is the same from any point of the space and is close to 1, the maximum value. Berestycki and Durrett studied speed of transposition and reversal random walks with the related edit distances while in the latter they used "approximate reversal distance" instead of reversal itself, ignoring the effect of hurdles and fortresses. This turns out to be the same as DCJ distance on single chromosomes. We have

(9) $$d_{r}\left(\pi ,\;I\right)=n+1-c\left(\pi \right)+h\left(\pi \right)+\tilde{f}\left(\pi \right)$$

while

(10) dcj(π,I)=n+1-c(π),

where *h*(*π*) and $\tilde{f}\left(\pi \right)$ are the number of hurdles and fortresses, respectively.

Although Berestycki and Durrett only proved their theorem for the random transposition r.w. on *S_n_*, they suggested that same method should carry over to reversal r.w. The following proposition is proved in [13] for *approximate *reversal distance (i.e., DCJ distance).

In this result and in the ensuing discussion *a_n _*is an arbitrary sequence such that *a_n _*→ ∞ as *n *→ 0. When it is unambiguous we drop *n *from $A_{t}^{\left(n\right)}$ and $X_{t}^{n}$.

**Propostition 1 ***[Berestycki-Durrett] Let c be fixed and let Xt be a reversal r.w. on *$S_{n}^{\pm }$*starting at id. Then*

(11) $$dcj\text{(}id\text{, }X_{cn\text{/2}}\text{)}=\text{(1}-f\text{(}c\text{))}n+w\text{(}n\text{),}$$

where

(12) $$f\left(c\right):=\frac{1}{c}\overset{\infty }{\underset{k=1}{\sum }}\frac{k^{k-2}}{k!}{\left(ce^{-c}\right)}^{k}$$

*and *$\frac{w\left(n\right)}{a_{n}\sqrt{n}}\to 0$*in probability*.

**Remark 1 ***The function *1 - *f is linear for c <*1, *f *(*c*) = 1 - *c*/2, *and sublinear for c >*1, 1 - *f *(*c*) *< c*/2 *This means that for c *≤ 1

(13) $$dcj\left(id,\;X_{cn/2}\right)-\frac{cn}{2}=w\left(n\right)$$

*and r.w. travels on an approximate geodesic (or parsimonious path) asymptotically almost surely. f is the function counting the number of tree components of an Erdös-Renyi random graph with n vertices for which the probability of having each edge is *$\frac{c}{n}$, *denoted by G*(*c, n*). *See Theorem 12 in *[14], *Chapter V*.

We extend the above theorem for the bonafide reversal distance. To do so we need to estimate the number of hurdles of $X_{\frac{cn}{2}}.$ Recall that an oriented cycle in a breakpoint graph is a cycle including an orientation edge, that is a grey edge with two black adjacency edges *e*, *e*', where a reversal involving *e *and *e*' splits the cycle [15]. As we discussed this is equivalent to saying *e * ∦ *e*'. It is not difficult to show

**Lemma 1 ***Let C *∈ *cBP*(π), *then C is oriented if and only if there exists exactly two equivalence classes of black edges, that is there exist at least two black edges with different signs*.

Then

**Theorem 1 ***Let c >*0 *be fixed and let X_t _be a reversal r.w.starting at id. Define h_t _*:= *h*(*X_t_*) *to be the number of hurdles in BP *(*X_t_*). *Then*

(14) $$\frac{h_{cn/2}}{a_{n}\sqrt{n}}\to 0\;in\;probability.$$

**Proof**. Cycles of the BP that have never been involved in a fragmentation event must be oriented, since the two rejoined black edges resulting from an inversion-induced merger of cycles cannot be parallel.

Therefore we need only to count the number of edges that have been involved in a fragmentation event. To do so we apply the method of counting cycles in [13], Theorem 3. Hurdles occur only in those cycles with length more than one that have been involved in a fragmentation up to time $\frac{cn}{2}.$ We call such cycles fragmented cycles. The number of fragmented cycles with length more than $\sqrt{\;n}$ is always less than $\sqrt{\;n}$. But to count all fragmented cycles in $X_{\frac{cn}{2}}$ with size less than $\sqrt{\;n}$ we need to find an upper bound for the rate of a fragmentation up to time $\frac{cn}{2}$. Since a fragmentation occurs when two black edges in one cycle are chosen, to fragment a cycle in BP, for any chosen black edge *e *we only can pick another black edge *e*' in the same cycle whose graph distance in the breakpoint graph is less than $2\sqrt{n}$. (The coefficient 2 arises from the fact that the cycles are alternating in BP.)

Thus the rate of fragmentation at an arbitrary time *t *is not more than $\frac{n}{n}\cdot \frac{2\left(\sqrt{n}\right)}{n}\;=\frac{2}{\sqrt{n}}$. Integrating up to time *t*, this gives us the expected number of fragmented cycles at time *t *is $\frac{2}{\sqrt{n}}\cdot t$. For $t=\frac{cn}{2}$ this expectation is $c\sqrt{n}.$ Now, dividing by $a_{n}\sqrt{n}$, the result follows from Chebyshev's inequality and the fact that hurdles only occurs in fragmented cycles. ■

**Theorem 2 ***let c >*0 *be fixed and let X_t _be a reversal r.w. on $S_{n}^{\pm }$ starting at id and let $d_{r}:=d_{r}^{\left(n\right)}$ denote the reversal distance on $S_{n}^{\pm }$. Then*

(15) $$d_{r}\left(id,\;X_{cn/2}\right)=\left(1-f\left(c\right)\right)n+w^{\prime }\left(n\right)$$

*where f is the same function as in the statement of Proposition 1 and w*' (*n*) *is a function with *$\frac{w^{\prime }\left(n\right)}{a_{n}\sqrt{n}}\to 0$*in probability*.

**Proof**. Since *d_r_*(Π) = *dcj*(Π) + *h*(Π) + *f*˜(Π) by the proposition we have *d_r _*(*X_cn/2_*) = (1 *− f *(*c*))*n *+ *w*(*n*) + *h_cn/2 _*+ *f*˜(*X_cn/2_*). But

(16) $$\frac{w^{\prime }\left(n\right)}{a_{n}\sqrt{n}}\;:=\frac{w\left(n\right)+h_{cn/2}+\tilde{f}\left(X_{cn/2}\right)}{a_{n}\sqrt{n}}\to 0$$

in probability, by the convergence of $\frac{w\left(n\right)}{a_{n}\sqrt{n}}$ and $\frac{h_{cn/2}}{a_{n}\sqrt{n}}$ in Proposition 1 and Theorem 2 and $\overset{\sim }{f}\left(X_{cn/2}\right)\;\leq 1.■$

**Theorem 3 ***Let *$X_{t}^{1,n},\;\ldots ,\;X_{t}^{k,n}$*be k independent reversal r.w in *$S_{n}^{\pm }$*starting at id. Suppose either*

*a) d *:= *dcj dcj distance*

or

*b) *$d:=d_{r}^{\left(n\right)}$*reversal distance*.

Then for c <$\frac{1}{4}$*we have *$\frac{\varepsilon_{{}^{c_{n}}}^{d,n}}{a_{n}\sqrt{n}}\to 0$*in probability*.

**Proof**. We prove the theorem only for *d_r_*. The proof of the DCJ case is similar. For all *i*, *j *∈ {1, ..., *k*} and for a median solution *x *of $A_{t}^{\left(n\right)}$

(17) $$d_{r}^{\left(n\right)}\left(X_{t}^{i,n},\;X_{t}^{j,n}\right)\leq d_{r}^{\left(n\right)}\left(x,\;X_{t}^{i,n}\right)+d_{r}^{\left(n\right)}\left(x,\;X_{t}^{j,n}\right).$$

Therefore,

(18) $$\underset{i\neq j}{\sum }d_{r}^{\left(n\right)}\left(X_{t}^{i,n},\;X_{t}^{j,n}\right)\leq \underset{i\neq j}{\sum }\left(d_{r}^{\left(n\right)}\left(x,\;X_{t}^{i,n}\right)+d_{r}^{\left(n\right)}\left(x,\;X_{t}^{j,n}\right)\right).$$

We conclude

(19) $$\sum d_{r}^{n}\left(X_{t}^{i,n},\;X_{t}^{j,n}\right)\leq \left(k-1\right)m^{d,n}\left(A_{t}^{\left(n\right)}\right)\leq \left(k-1\right)g_{A_{t}^{\left(n\right)}}\left(id\right).$$

Let $c\leq \frac{1}{4}$. Then by Theorem 2 we have for all *i, j i *≠ *j*

(20) $$d_{r}^{\left(n\right)}\left(X_{cn}^{i,n},\;X_{cn}^{j,n}\right)=2cn-w\left(n\right)$$

and

(21) $$d_{r}^{\left(n\right)}\left(id,\;X_{cn}^{i,n}\right)=cn-w\left(n\right)$$

where $\frac{w\left(n\right)}{\left(a_{n}\sqrt{n}\right)}\to 0$ in probability. Thus

(22) $$\left(\begin{array}{c} k\\ 2 \end{array}\right)\left(2cn\;-w\left(n\right)\right)\leq \left(k-1\right)m^{d,n}\left(A_{cn}^{\left(n\right)}\right)\leq \left(k-1\right)k\left(cn\;-w\left(n\right)\right).$$

Then

(23) $$|m^{d,n}\left(A_{cn}^{\left(n\right)}\right)-kcn|\;\leq k^{\prime }w\left(n\right)$$

for a constant *k*'. Also $|g_{A_{cn}^{\left(n\right)}}\left(id\right)-kcn|\;\leq kw\left(n\right)$. Therefore, there exists a constant k* such that

(24) $$|m^{d,n}\left(A_{cn}^{\left(n\right)}\right)-g_{A_{cn}^{\left(n\right)}}^{d,n}\left(id\right)|\;\leq k^{*}w\left(n\right).$$

This implies

(25) $$\frac{\varepsilon_{cn}}{a_{n}\sqrt{n}}=\frac{m^{d,n}\left(A_{cn}^{\left(n\right)}\right)-g_{A_{cn}^{\left(n\right)}}^{d,n}\left(id\right)}{a_{n}\sqrt{n}}\to 0\;in\;probability.$$

This proves the theorem. ■

**Remark 2 ***The statement of the theorem suggests ignoring the error of order *$o\left(a_{n}\sqrt{n}\right)$*for a_n _*→ ∞. *id remains as the median of leaves of k independent stochastic processes *$X_{t}^{1,n},\;\ldots ,\;X_{t}^{k,n}$*up to time *$\frac{n}{4}$*asymptotically almost surely*.

**Theorem 4 ***Let *$c\;\leq \frac{1}{4}$*be fixed. Suppose d is either DCJ or reversal distance. Then by the hypothesis of Theorem 3*

(26) $$\frac{kcn-m^{d,n}\left(A_{cn}\right)}{a_{n}\sqrt{n}}\to 0\;in\;probability\;as\;n\to \infty .$$

**Proof**. This follows directly from the fact that

(27) $$\frac{kcn-g_{A_{cn}}^{d,n}\left(id\right)}{a_{n}\sqrt{n}}\to 0$$

in probability. ■

Now, it is natural to ask whether the statement of Theorem 4 also holds for some time after $\frac{n}{4}$. In other words, is the median value *kcn *a fair estimator for the total time of divergence? We conjecture not, that the property is lost after time $\frac{n}{4}$, but for now can only prove a weaker upper bound for this time.

**Theorem 5 ***Let *$c>\frac{1}{2}$*be fixed. Suppose d is either DCJ or reversal distance. Then by the same hypothesis as in Theorem 3*

(28) $$\frac{kcn-m^{d,n}\left(A_{cn}\right)}{n}\to \alpha_{c}$$

where

(29) $$\alpha_{c}:=k\left(1-f\left(2c\right)\right)$$

*is strictly positive for *$c>\frac{1}{2}$

**Remark 3 ***This theorem shows after time *$\frac{n}{2}$*the error is of order n and so the median value is not a good estimate of k times the divergence time*.

Proof.

(30) $$kcn-m^{d,n}\left(A_{cn}\right)\geq kcn-g_{A_{cn}}^{d,n}\left(id\right)=k\left(1-f\left(2c\right)\right)n+w\left(n\right),$$

where $\frac{w\left(n\right)}{a_{n}\sqrt{n}}\to 0$ in probability. Dividing by *n*, the result follows. ■

In fact, since *f *(*c*), *c >*0 is decreasing and for *c <*1, $f\left(c\right)=1-\frac{c}{2}$, it is easy to see that in the case *k *= 3, for *c >*0.75, $\varepsilon_{cn}^{d,n}$ is of order $\beta_{c}^{d}n$ for some $\beta_{c}^{d}\geq 0$.

**Theorem 6 ***Let k *= 3 and d be either dcj or dr. Consider the same hypothesis in Theorem 3. Assume c* be solution of

(31) $$f\left(\frac{x}{2}\right)=\frac{1}{3}.$$

Then for all c > c* *there exists *$\beta_{c}^{d}$*such that*

(32) $$\varepsilon_{\frac{cn}{4}}^{d,n}=o\left(\beta_{c}^{d}n\right).$$

Proof.

(33) $$m^{d,n}\left(A_{\frac{cn}{4}}\right)\leq d\left(X_{\frac{cn}{4}}^{1,n},\;X_{\frac{cn}{4}}^{2,\;n}\right)+d\left(X_{\frac{cn}{4}}^{1,\;n},\;X_{\frac{cn}{4}}^{3,\;n}\right).$$

Computing $d\left(X_{\frac{cn}{4}}^{1,\;n},\;X_{\frac{cn}{4}}^{i,n}\right)$ for *i *= 2, 3 is the same as $d\left(id,\;X_{\frac{cn}{2}}^{1,\;n}\right)$. This is true since the Cayley graph of $S_{n}^{\pm }$ w.r.t. reversals is symmetric and regular and so $P\left(X_{0}=id,\;X_{\frac{cn}{4}}=\Pi \right)=P\left(X_{0}=\Pi ,\;X_{\frac{cn}{4}}=id\right)$. But therefore by symmetry of the Cayley graph we can just consider $d\left(id,\;X_{\frac{cn}{2}}^{1,\;n}\right)$. Hence,

(34) $$m^{d,n}\left(A_{\frac{cn}{4}}\right)\leq 2\left(1-f\left(c\right)\right)n+2w\left(n\right).$$

Let *x >*0 be so that

(35) $$0<-2\left(1-f\left(x\right)\right)+3\left(1-f\left(\frac{x}{2}\right)\right).$$

This means

(36) $$g_{A_{\frac{cn}{4}}}^{d,n}\left(id\right)>m^{d,n}\left(A_{\frac{cn}{4}}\right).$$

So it suffices to prove above inequality for *x *= *c *>*c*^∗^. Since *f *(*x*) *>*0 for all *x >*0

(37) $$1+2f\left(x\right)-3f\left(\frac{x}{2}\right)>1-3f\left(\frac{x}{2}\right)$$

in which the right hand side is strictly increasing, Therefore for all *c *≥ c*

(38) $$1+2f\left(c\right)-3f\left(\frac{c}{2}\right)>1-3f\left(\frac{c^{*}}{2}\right)=0.$$

This proves the statement. ■

Now, we would like to measure the volume of that part of the space $S_{n}^{\pm }$ for which median does well, compared with the whole space. The ratio of the two converges to 0 as *n *goes to *∞*, showing that the median is only useful in a highly restricted region of the space.. The following theorem is entailed by a theorem in [16]. Let *c_n _*= *c_n_*(Π) be the number of cycles in the BP graph of a random $\Pi \in S_{n}^{\pm }$. Let *d_n_*be a distance (metric) on $S_{n}^{\pm }$. Define

(39) $$B_{cn}^{d}=B_{cn}^{d,n}:=\left\{\Pi \in S_{n}^{\pm },\;d\left(\Pi ,\;id\right)\leq cn\right\}$$

to be the ball of radius *cn *in $S_{n}^{\pm }$.

**Theorem 7 ***Let *0 *< c <*1 *be fixed. Then*

(40) $$a)\gamma_{n}=\frac{\left|B_{cn}^{dcj}\right|}{\left|S_{n}^{\pm }\right|}\to 0\;as\;n\to \infty ,$$

(41) $$b)\gamma {'}_{n}=\frac{\left|B_{cn}^{d_{r}}\right|}{\left|S_{n}^{\pm }\right|}\to 0\;as\;n\to \infty .$$

Proof.

a)

(42) $$For\;all\;\Pi \in B_{cn}^{dcj}\;,\;|cBP\left(\Pi \right)|\;\geq \left(1-c\right)n.$$

Suppose γ*_n _*does not converge to 0. Therefore there exists a subsequence ${\left\{n_{i}\right\}}_{i\in \mathbb{N}}$ such that $\gamma_{n_{i}}\geq \varepsilon$ for a constant *ε >*0. This implies

(43) $$E\left(c_{n_{i}}\right)\geq \varepsilon \left(1-c\right)n_{i}.$$

But by Theorem 2.2 in [16], we have

(44) $$\frac{E\left(c_{n_{i}}\right)}{n_{i}}\to 0\;\;as\;\;n_{i}\to \infty .$$

That is in contradiction with the above inequality since

(45) $$\frac{\varepsilon \left(1-c\right)n_{i}}{n_{i}}\to \varepsilon \left(1-c\right)>0.$$

b) For the second part it suffices to observe that for all $\Pi \in S_{n}^{\pm }$ we have

(46) $$d_{r}\left(\Pi \right)\geq dcj\left(\Pi \right).$$

Therefore

(47) $$B^{d_{r}}\left(\Pi \right)\subset B^{dcj}\left(\Pi \right)$$

and the result follows part (a) since

(48) $$\gamma_{n}^{\prime }\leq \gamma_{n}\to 0\;as\;n\to \infty .$$

■

## Conclusion

We have shown that the median value for DCJ and for reversal distance for a reversal r.w.has good limiting properties if the number of steps remains below *cn*/4, for any *c <*1, but for some value *c *> 1, more than this number of steps destroys these limiting properties. The critical value may indeed be *c *= 1, but for now we can only show that for *c *> 3 (and *c *> 2) the median value is no longer a good estimator of the distance between the *id *and the current position of the r.w. (and *k *times the divergence time, respectively).

Note that a simulation strategy to estimate *c *is not available because of the hardness of calculating the median. As *n *increases even to moderate values all exact methods require prohibitive computing time.

These results imply that the steinerization strategy for the small phylogeny problem may lead to poor estimates of the interior nodes of a phylogeny unless the taxon sampling is sufficient to assure that a "similar genomes condition" holds for every k-tuple of genomes used in the course of of the iterative optimization search. This can be monitored prior to each step in the iterative optimization of the phylogeny through successive application of the median method.

## Competing interests

The authors declare that they have no competing interests.

## Authors' contributions

AJ and DS planned the study, carried out the research and wrote the article.

## Declarations

Publication of this article was supported by the Canada Research Chair in Mathematical Genomics.

This article has been published as part of *BMC Bioinformatics *Volume 14 Supplement 15, 2013: Proceedings from the Eleventh Annual Research in Computational Molecular Biology (RECOMB) Satellite Workshop on Comparative Genomics. The full contents of the supplement are available online at http://www.biomedcentral.com/bmcbioinformatics/supplements/14/S15.
